# Investigation into the restoration of TRPM3 ion channel activity in post-COVID-19 condition: a potential pharmacotherapeutic target

**DOI:** 10.3389/fimmu.2024.1264702

**Published:** 2024-05-03

**Authors:** Etianne Martini Sasso, Katsuhiko Muraki, Natalie Eaton-Fitch, Peter Smith, Andrew Jeremijenko, Paul Griffin, Sonya Marshall-Gradisnik

**Affiliations:** ^1^ The National Centre for Neuroimmunology and Emerging Diseases, Menzies Health Institute Queensland, Griffith University, Gold Coast, QLD, Australia; ^2^ Consortium Health International for Myalgic Encephalomyelitis, National Centre for Neuroimmunology and Emerging Diseases, Menzies Health Institute Queensland, Griffith University, Gold Coast, QLD, Australia; ^3^ School of Pharmacy and Medical Sciences, Griffith University, Gold Coast, QLD, Australia; ^4^ Laboratory of Cellular Pharmacology, School of Pharmacy, Aichi-Gakuin University, Nagoya, Japan; ^5^ Clinical Medicine, Griffith University, Gold Coast, QLD, Australia; ^6^ Department of Medicine and Infectious Diseases, Mater Hospital and Mater Medical Research Institute, Brisbane, QLD, Australia

**Keywords:** post COVID-19 condition, long Covid, SARS-CoV-2, myalgic encephalomyelitis/chronic fatigue syndrome, calcium, transient receptor potential melastatin 3, naltrexone hydrochloride, whole-cell patch-clamp electrophysiology

## Abstract

**Introduction:**

Recently, we reported that post COVID-19 condition patients also have Transient Receptor Potential Melastatin 3 (TRPM3) ion channel dysfunction, a potential biomarker reported in natural killer (NK) cells from Myalgic Encephalomyelitis/Chronic Fatigue Syndrome (ME/CFS) patients. As there is no universal treatment for post COVID-19 condition, knowledge of ME/CFS may provide advances to investigate therapeutic targets. Naltrexone hydrochloride (NTX) has been demonstrated to be beneficial as a pharmacological intervention for ME/CFS patients and experimental investigations have shown NTX restored TRPM3 function in NK cells. This research aimed to: i) validate impaired TRPM3 ion channel function in post COVID-19 condition patients compared with ME/CFS; and ii) investigate NTX effects on TRPM3 ion channel activity in post COVID-19 condition patients.

**Methods:**

Whole-cell patch-clamp was performed to characterize TRPM3 ion channel activity in freshly isolated NK cells of post COVID-19 condition (*N* = 9; 40.56 ± 11.26 years), ME/CFS (*N* = 9; 39.33 ± 9.80 years) and healthy controls (HC) (*N* = 9; 45.22 ± 9.67 years). NTX effects were assessed on post COVID-19 condition (*N* = 9; 40.56 ± 11.26 years) and HC (*N* = 7; 45.43 ± 10.50 years) where NK cells were incubated for 24 hours in two protocols: treated with 200 µM NTX, or non-treated; TRPM3 channel function was assessed with patch-clamp protocol.

**Results:**

This investigation confirmed impaired TRPM3 ion channel function in NK cells from post COVID-19 condition and ME/CFS patients. Importantly, PregS-induced TRPM3 currents were significantly restored in NTX-treated NK cells from post COVID-19 condition compared with HC. Furthermore, the sensitivity of NK cells to ononetin was not significantly different between post COVID-19 condition and HC after treatment with NTX.

**Discussion:**

Our findings provide further evidence identifying similarities of TRPM3 ion channel dysfunction between ME/CFS and post COVID-19 condition patients. This study also reports, for the first time, TRPM3 ion channel activity was restored in NK cells isolated from post COVID-19 condition patients after *in vitro* treatment with NTX. The TRPM3 restoration consequently may re-establish TRPM3-dependent calcium (Ca^2+^) influx. This investigation proposes NTX as a potential therapeutic intervention and TRPM3 as a treatment biomarker for post COVID-19 condition.

## 1 Introduction

The term post-COVID-19 condition (also known as long COVID, post-acute sequelae of COVID-19, and post-COVID-19 syndrome) refers to long-term effects caused by severe acute respiratory syndrome coronavirus 2 (SARS-CoV-2) infection, persisting 3 months from onset, with symptoms fluctuating or relapsing over time and occurring independently of severity or duration of acute infection (1, 2). As of July 2023, there are more than 768 million SARS-CoV-2 confirmed cases and approximately 30% of these people may develop post-COVID-19 condition without return to their prior state of health (3–5). Mortality rates of COVID-19 have decreased with the advent of vaccination; however, post-COVID-19 condition has emerged as a significant worldwide public health challenge (6–11).

Patients experiencing post-COVID-19 condition present a range of abnormalities affecting multiple systems including neurocognitive, immunological, gastrointestinal, and cardiovascular manifestations (7, 10, 12–14). A recent systematic review and meta-analysis by Lopez-Leon et al. identified over 50 long-term effects associated with post-COVID-19 condition, reflecting its diverse symptomatology (14). Common symptoms reported by post-COVID-19 condition patients include fatigue, post-exertional malaise, pain, dyspnea, cognitive impairment, and sleep disorders, symptoms that overlap with myalgic encephalomyelitis/chronic fatigue syndrome (ME/CFS) (15–21). Another similar aspect to ME/CFS is that post-COVID-19 condition patients also have exacerbating symptoms following physical or mental stress (10, 22–25).

ME/CFS is a complex multisystemic condition characterized by post-exertional neuroimmune exhaustion (PENE) and hallmarked by chronic fatigue that is not alleviated by rest (25–28). Resembling post-COVID-19 condition, no diagnostic test is available for ME/CFS, and it follows the fulfilment of diagnostic case definitions; the exclusion of other conditions may account for the symptoms. Although ME/CFS etiology has been considered to be not clearly understood, post-infectious onset, dysfunction of the immune system with reduced natural killer (NK) cell cytotoxic activity, impaired calcium (Ca^2+^) mobilization, and the dysfunction of transient receptor potential melastatin 3 (TRPM3) ion channels are associations to ME/CFS supported by literature (29–36).

TRPM3 is a non-selective ion channel highly permeable to Ca^2+^ and broadly expressed in the human body including the brain, spinal cord, kidney, liver, pancreas, cardiovascular organs, skeletal muscle, genitourinary, testis, ovary, and immune cells (32, 37–41). The contribution of TRPM3 is associated with diverse physiological processes, such as substantial regulatory function in Ca^2+^ homeostasis that leads to intracellular signaling pathways, cell differentiation, adhesion, division and apoptosis, transcriptional events, immune synapse formation, degranulation, the release of cytolytic proteins, and so on (32, 42–44). Furthermore, TRP ion channels may facilitate host–viral interactions through the regulation Ca^2+^ and may promote viral pathogenesis during SARS-CoV-2 infection and, therefore, provide a target for anti-viral therapeutics (45–47).

Dysregulation of TRPM3 interferes with intracellular Ca^2+^ mobilization and consequently affects intracellular signaling pathways, which has previously been demonstrated in NK cells from ME/CFS patients (31, 34, 48, 49). As a significant decrease in NK cell cytotoxicity is consistently reported in ME/CFS, NK cells provide an appropriate and non-invasive cell model to understand the pathomechanism and disease progression of ME/CFS and post-COVID-19 condition (50–52). Despite the suggestion of an overlap between post-COVID-19 condition and ME/CFS, only recently did a laboratory-based investigation corroborate this hypothesis (53). Our recent study suggested that NK cells from post-COVID-19 condition patients also had TRPM3 ion channel dysfunction similar to ME/CFS patients. In fact, TRPM3 activity by pregnenolone sulfate (PregS), a TRPM3 agonist, in NK cells from post-COVID-19 condition patients mimicked the results from ME/CFS patients, while a significant difference was found between post-COVID-19 condition patients and healthy controls (HC). In addition, NK cells from ME/CFS and post-COVID-19 condition groups were resistant to ononetin, a TRPM3 inhibitor, in the presence of PregS (53). Similarities between post-COVID-19 condition and ME/CFS patients suggest a common pathophysiology that should be investigated to understand and improve the symptoms of both conditions (54).

Currently, there is limited evidence-based treatment to alleviate the spectrum of symptoms experienced by post-COVID-19 condition patients. However, growing evidence suggests the *in vitro* effects of naltrexone hydrochloride (NTX) on ME/CFS and that low-dose NTX (LDN) is a safe and effective pharmaceutical option for ME/CFS and other chronic diseases (34, 49, 55). NTX is a Mu (μ)-opioid receptor (OR) antagonist that negates the inhibitory function μOR on TRPM3 ion channels. The signaling pathways involved in NTX restoration of TRPM3 channel functions are the inhibition of G-protein coupled receptors (GPCRs) and, consequently relief of TRPM3 inhibited by the G-protein subunit Gβγ (49, 56). Given that the activity of TRPM3 ion channels is modulated by Gβγ subunits, it is hypothesized that inhibition of µOR with NTX restores TRPM3 ion channel function and may consequently re-establish TRPM3-dependent Ca^2+^ influx in NK cells (34, 49, 56). ORs are broadly distributed in tissues and organ systems, including immune cells; thus, opioids have immunomodulatory and immunosuppressive effects (57).

In this investigation, we aimed to validate our previous study of impaired TRPM3 channel in NK cells from post-COVID-19 condition and ME/CFS participants. In addition, we also intended to further investigate the potential effects of *in vitro* treatment with NTX on TRPM3 dysfunction in NK cells from post-COVID-19 condition patients.

## 2 Materials and methods

### 2.1 Recruitment

The National Centre for Neuroimmunology and Emerging Diseases (NCNED) patient database and clinician referral were used to select nine post-COVID-19 condition, nine ME/CFS, and nine HC participants from February 2022 to April 2023. All participants were aged between 18 and 65 years. To be included in the study, patients with post-COVID-19 condition and ME/CFS must have had a previous medical diagnosis and screened through an online questionnaire corresponding with the Fukuda [Centers for Disease Control and Prevention (CDC)] (58), Canadian Consensus Criteria (CCC) (59), and International Consensus Criteria (ICC) (25). All post-COVID-19 condition patients met clinical case definition by Delphi consensus from the World Health Organization (WHO) (2), while HC participants were in good health without incidence of fatigue or evidence of illness. All HC and ME/CFS participants were without a previous history of COVID-19 infection and had never had a positive COVID-19 test, polymerase chain reaction (PCR) test, or rapid antigen test (RAT) result, nor had they been suspected of having SARS-CoV-2 infection. Any volunteer that had suspected COVID-19 infection symptoms but was not tested was excluded from this study.

Participants were excluded from this study if they were pregnant, breastfeeding, or reported previous alcohol abuse or chronic illness (such as autoimmune diseases, cardiovascular disease, diabetes, metabolic syndrome, thyroid disease, malignancies, insomnia, chronic fatigue, and primary psychological disorders) or obese [body mass index (BMI) ≥ 30]. The use of opioids or any other pain killers in the previous 3 months or pharmacological agents that directly or indirectly influence TRPM3 or Ca^2+^ signaling was also an exclusion criterion applied in this study. Participants were provided with the option to cease any conflicting medications for a minimum of 14 days prior to blood donation after approval from their physician. An online questionnaire was completed by all participants to obtain sociodemographic background, medical history, and medications taken, and for post-COVID-19 condition and ME/CFS participants, the questionnaire also included symptom history.

Symptoms were categorized in 10 different groups including cognitive difficulties; pain; sleep disturbances; cardiovascular, gastrointestinal, respiratory, urinary, immunological, and thermostatic intolerances; and sensory disturbances. The 36-item short form health survey (SF-36) was used to assess participants’ quality of life (QoL) and items within the same domain were combined into eight scale scores, namely, physical functioning, role limitations due to physical health problems, bodily pain, general health perceptions, energy/vitality, social functioning, role limitations due to personal or emotional problems, and emotional wellbeing/general mental health. Domains were scored on a scale of 0% to 100%, whereby higher QoL was indicated by high scores (60).

The WHO Disability Assessment Schedule (DAS) was used to evaluate disability. This reported outcome measure assessed functional capacity across six domains of life: cognition, mobility, self-care, getting along, general life activities, and participation in society. The Life Activities domain specific to work or school was not included in the analysis given that many people suffering from ME/CFS or post-COVID-19 condition report being unemployed. Each of the 36 items of the WHO DAS 2.0 was scored on a five-point scale: none, mild, moderate, severe, and extreme or cannot do. The subscale scores were generated by first converting each item score into the corresponding, pre-defined weighted values as outlined in the WHO DAS 2.0 manual (61). Low percentage scores were associated to no disability while full disability was represented by 100% (62).

This research was approved by the Griffith University Human Research Ethics Committee (GU HREC 2022/666).

### 2.2 NK cell isolation

A blood sample of 84 mL was collected in ethylenediaminetetraacetic acid (EDTA) tubes from each participant via venepuncture. All blood collections were performed between 6:30 a.m. and 10:30 a.m. at convenient locations for participants, including the Gold Coast campus of Griffith University, Robina Hospital, patient homes, or private laboratories in South East Queensland and North East New South Wales. All participants performed a RAT on the collection day to check that no one was infected with SARS-CoV-2 when they donated blood. Full blood count was performed within 4 h of collection for each participant to analyze red blood cell count, white blood cell count, and granulocyte cell count at Gold Coast University Hospital or private laboratories in Australia.

Samples were provided to the laboratory de-identified using a unique alphanumeric code. A total of 80 mL of whole blood was used to isolate peripheral blood mononuclear cells (PBMCs), as previously described (50, 63). PBMCs count and cell viability were determined using trypan blue stain (Invitrogen, Carlsbad, CA, USA) and an automatic cell counter (TC20 Automated cell counter, Bio-Rad, Laboratories, Hercules, CA).

Consecutively, a commercial kit, EasySep Negative Human NK Cell Isolation (Stem Cell Technologies, Vancouver, BC, Canada), was used to isolate NK cells by immunomagnetic selection. Flow cytometry was used to determine NK cell purification, and experiments were performed as previously described (30). The phenotypic surface expression CD3^−^CD56^+^ was used to identify NK cell population. Samples with NK cells purity ≥ 75% were included in this study. **Supplementary Figure 1** represents NK cell purity results from HC, ME/CFS, and post-COVID-19 condition patients, without statistical difference between groups.

### 2.3 Whole-cell electrophysiological recording

The TRPM3 ion channel activity of NK cells from post-COVID-19 condition, ME/CFS, and HC participants was characterized by the patch-clamp technique, as previously described (31, 48, 49, 53, 56). Briefly, we performed electrophysiological recordings using borosilicate glass capillary electrodes (outside diameter = 1.5 mm; inside diameter = 0.86 mm, Harvard Apparatus, Holliston, MA, USA). When filled with pipette solution, the pipette resistance was 8–12 MΩ. A CV203BU head-stage (Molecular Devices, Sunnyvale, CA, USA) was used to mount pipettes and it connected to a three-way coarse manipulator and a micro-manipulator (Narishige, Tokyo, Japan). Axopatch 200B amplifier and pClamp 10.7 software (Molecular Devices, Sunnyvale, CA, USA) were used to amplify and record electrical signals. Data were filtered at 5 kHz and sampled digitally at 10 kHz via a Digidata 1440A analogue-to-digital converter (Molecular Devices, Sunnyvale, CA, USA). The voltage-ramp protocol was a step from a holding potential of +10 mV to −90 mV, followed by a 0.1-s ramp to +110 mV, before returning to +10 mV (repeated every 10 s), with correction of liquid junction potential between the pipette and bath solutions (−10 mV) and leak current component was not subtracted from the recorded currents. Intracellular pipette solution and pipette solution were composed, prepared, and stored as previously described (31). L-aspartic acid was included in the intracellular pipette solution to reduce the possibility of chloride current being involved in recordings.

Pharmacological modulators were added in the bath solution to identify TRPM3 ionic currents as previously validated by NCNED (31), using 100 μM PregS (Tocris Bioscience, Bristol, UK) and later blocking with 10 μM ononetin in the presence of PregS (Tocris Bioscience, Bristol, UK). Experiments were performed at room temperature (20–25°C). In addition, authors excluded from the analysis any cells with unstable currents or Cl- contamination. The protocol for TRPM3 recording consists in running extracellular perfusion solution (bath solution) for 50 s to establish a baseline current, followed by 100 μM PregS for 2.5 min and subsequently 10 μM ononetin and 100 μM PregS for 2.5 min. After drug application, cells are washed with a bath solution for 100 s. In our perfusion system, each drug concentration in the recording chamber reached maximum within 60 s. All records were analyzed individually through the process explained in **Supplementary Figure 2**.

### 2.4 *In vitro* treatment

In this study, freshly isolated NK cells, at a concentration of 2×10^5^ cells/mL, were incubated for 24 h at 37°C with 5% CO_2_ in RPMI-1640 with no phenol and with L-glutamine supplemented with 10% fetal bovine serum (FBS) and 20 IU/mL of recombinant human interleukin-2 (IL-2) (specific activity 5×10^6^ IU/mg) (Miltenyi Biotec, BG, Germany) in two different protocols: (A) non-treated (control) and (B) treated with 200 µM NTX (Sigma-Aldrich, St. Louis, MO, USA). Endotoxin-free water was used to resuspend NTX just before each incubation. After 24 h of incubation, cells were prepared for patch-clamp experiments using the same protocol of freshly isolated NK cells (baseline).

Part of the NK cells from seven HC and nine post-COVID-19 condition participants were incubated to determine effects of *in vitro* treatment with NTX in the restoration of TRPM3 on cells from post-COVID-19 condition patients. Because of an insufficient NK cell count from *N* = 2 HC, cells from seven HC were included in the incubation with and without NTX. The complete design of this study is presented in **Supplementary Figure 3**.

### 2.5 Statistical analysis

Flow cytometry data were exported from Accuri C6 software, and GraphPad Prism v9 (GraphPad Software Inc., Version 9, La Jolla, CA, USA) was used for purity comparison. Participant characteristics and full blood count parameters were analyzed using Statistical Package for the Social Sciences (SPSS) v26 (IBM Corp, Version 24, Armonk, NY, USA). Electrophysiological data were analyzed with pCLAMP 10.7 software (Molecular Devices, Sunnyvale, CA, USA), Origin 2021 (OriginLab Corporation, Northampton, MA, USA), and GraphPad Prism v9 (GraphPad Software Inc., Version 9, La Jolla, CA, USA). The ROUT method was used to identify and remove outliers from analysis. Histogram plots and the Shapiro–Wilk normality test were used to assess normality of distribution of parameters. The independent nonparametric Kruskal–Wallis (Dunn’s multiple comparisons) test or Mann–Whitney *U* test was performed for statistical comparison, according to the number of groups analyzed, while Fisher’s exact test (Bonferroni method) was applied to identify NK cells’ resistance to ononetin in the presence of PregS. Power calculations using G*Power are now included to support the sample size of the current manuscript (31, 34, 48, 49). Using the mean and SD from these studies, a sample size greater than *n* = 5 per participant per group achieves sufficient statistical power with the following parameters: (i) an effect size of 0.5, (ii) a type 1 error of 5% (α = 0.05), and (iii) a power of 80%. Significance was set at *p* < 0.05 and data are shown as mean ± SEM, unless otherwise stated.

## 3 Results

### 3.1 Participants

Twenty-seven participants were included in this investigation, distributed into three different groups, *N* = 9 HC, *N* = 9 post-COVID-19 condition patients, and *N* = 9 ME/CFS patients. All post-COVID-19 condition patients met WHO clinical case definition (2), and two of them also met the CCC for ME/CFS (59). One ME/CFS patient met ICC (25), two met CCC, and the remaining six met both clinical criteria. All ME/CFS and post-COVID-19 condition participants did not report other fatigue-related illnesses that explain their symptoms. Demographic data are presented in **Table 1**. The age average and standard derivation (SD) were 45.22 ± 9.67 years old for HC, 40.56 ± 11.26 years old for post-COVID-19 condition patients, and 39.33 ± 9.80 years old for ME/CFS patients. Seven HC, seven post-COVID-19 condition, and five ME/CFS were female participants. There were no significant differences in age, BMI, gender, or level of education among groups, but a significant difference was found in employment status (*p* = 0.002). Illness/Disability was more frequently reported in ME/CFS patients (77.8%) compared with HC and post-COVID-19 condition patients with full-time employment (both 66.7%).

**Table 1:** Participant demographics.

		HC	ME/CFS	Post-COVID-19 condition	*p*-value
Age (years)		45.22 ± 9.67	39.33 ± 9.80	40.56 ± 11.26	0.432
Gender, *N* (%)	Female Male	7 (77.8%) 2 (22.2%)	5 (55.6%) 4 (44.4%)	7 (77.8%) 2 (22.2%)	0.504
BMI (kg/m^2^)		24.03 ± 3.70	23.63 ± 4.26	25.58 ± 3.90	0.610
Employment status	Full time	6 (66.7%)	1 (11.1%)	6 (66.7%)	**0.002**
	Part time	1 (11.1%)	1 (11.1%)	2 (22.2%)	
	Casual	1 (11.1%)	0 (0.0%)	1 (11.1%)	
	Unemployed	1 (11.1%)	0 (0.0%)	0 (0.0%)	
	Illness/Disability	0 (0.0%)	7 (77.8%)	0 (0.0%)	
Education	Primary education	0 (0.0%)	0 (0.0%)	0 (0.0%)	0.191
	High school	1 (11.1%)	1 (11.1%)	2 (22.2%)	
	Undergraduate	1 (11.1%)	2 (22.2%)	5 (55.6%)	
	Postgraduate/Doctoral	6 (66.7%)	1 (11.1%)	0 (0.0%)	
	Other	1 (11.1%)	5 (55.6%)	2 (22.2%)	

Data presented as mean ± SD or N (%). Values of p < 0.05 are in boldface. HC, healthy controls; ME/CFS, myalgic encephalomyelitis/chronic fatigue syndrome; BMI, body mass index.

The QoL and disability from all participants was assessed using WHO DAS and SF-36 surveys, as shown in **Table 2**. Significant differences were identified in all domains of SF-36 scores between groups in domains of physical functioning, physical role, general health, energy/vitality and social functioning (*p* < 0.001), pain (*p* = 0.001), emotional role (*p* = 0.005), and emotional wellbeing (*p* = 0.006). SF-36 scores were significantly reduced in ME/CFS and post-COVID-19 condition participants compared with HC. SF-36 scores were comparable between ME/CFS and post-COVID-19 condition patients. All domains of WHO DAS also reported significant differences for mobility, communication and understanding, life activities, participation in society (*p* < 0.001), self-care (*p* = 0.009), and interpersonal relationships (*p* = 0.001). ME/CFS and post-COVID-19 condition patients reported significantly higher disability in all WHO DAS domains compared with HC. WHO DAS scores were comparable between ME/CFS and post-COVID-19 condition. All participants had full blood count parameters in the normal range, and a significant difference was only detected in platelets between groups (*p* = 0.032).The average age of illness onset and SD were 23.78 ± 9.35 years old for ME/CFS patients and 40.11 ± 11.14 years old for post-COVID-19 condition patients, while disease duration was 15.56 ± 9.67 years for ME/CFS and 0.37 ± 0.14 years for post-COVID-19 condition patients. All ME/CFS patients declared cognitive difficulties, pain, and sleep disturbances, while cognitive difficulties were experienced by 7 (77.8%), and pain and sleep disturbances were experienced by 8 (88.9%) post-COVID-19 condition patients. Similarly, sensory disturbances were declared by 8 (88.9%) post-COVID-19 condition and 7 (77.8%) ME/CFS patients. Immune disturbances were reported by 7 (77.8%) and thermostatic instability was reported by 6 (66.7%) participants from each group. All data of symptoms are detailed in **Table 3** and data on frequency and severity of key symptoms are attached as a **Supplementary Material** (**Supplementary Table 1**).

**Table 2:** Participant quality of life, disability scores, and serology.

	HC	ME/CFS	Post-COVID-19 condition	*p*-value
SF-36 (%)				
Physical functioning	100.0 ± 0.0	37.78 ± 32.51	57.22 ± 33.83	**<0.001**
Physical role	100.0 ± 0.0	11.82 ± 17.54	37.51 ± 32.32	**<0.001**
Pain	97.78 ± 4.41	46.94 ± 29.71	50.00 ± 29.32	**0.001**
General health	77.79 ± 11.99	31.94 ± 19.66	49.34 ± 21.19	**<0.001**
Social functioning	91.67 ± 17.68	23.61 ± 25.34	33.33 ± 32.48	**<0.001**
Emotional role	94.44 ± 13.83	63.89 ± 27.95	46.29 ± 33.37	**0.005**
Emotional wellbeing	82.78 ± 20.48	52.22 ± 16.98	50.56 ± 23.51	**0.006**
Vitality	78.49 ± 21.69	12.51 ± 18.48	18.78 ± 12.51	**<0.001**
WHO DAS (%)				
Communication and understanding	4.17 ± 12.50	53.71 ± 21.80	31.48 ± 26.44	**<0.001**
Mobility	0.0 ± 0.0	53.89 ± 32.67	31.11 ± 22.05	**<0.001**
Self-care	0.0 ± 0.0	38.91 ± 39.25	11.81 ± 18.35	**0.009**
Interpersonal relationships	2.78 ± 8.33	51.41 ± 29.60	32.66 ± 34.07	**0.001**
Life activities	0.0 ± 0.0	68.76 ± 28.30	43.73 ± 30.92	**<0.001**
Participation in society	3.82 ± 9.34	61.12 ± 29.04	43.77 ± 21.54	**<0.001**
Full blood count				
White cell count (4.0–11.0 × 10^9^/L)	5.98 ± 0.92	5.27 ± 0.72	5.80 ± 1.10	0.273
Lymphocytes (1.0–4.0 × 10^9^/L)	1.86 ± 0.36	1.90 ± 0.35	1.82 ± 0.30	0.879
Neutrophils (2.0–8.0 × 10^9^/L)	3.42 ± 0.62	2.82 ± 0.65	3.31 ± 1.13	0.210
Monocytes (0.1–1.0 × 10^9^/L)	0.46 ± 0.13	0.38 ± 0.06	0.46 ± 0.17	0.289
Eosinophils (<0.6 × 10^9^/L)	0.18 ± 0.13	0.13 ± 0.11	0.17 ± 0.18	0.704
Basophils (<0.2 × 10^9^/L)	0.05 ± 0.02	0.03 ± 0.01	0.03 ± 0.01	0.055
Platelets (140–400 × 10^9^/L)	274.2 ± 43.45	226.6 ± 34.46	277.1 ± 42.92	**0.032**
Red cell count (3.8–5.2 × 10^12^/L)	4.64 ± 0.37	4.63 ± 0.39	4.60 ± 0.38	0.945
Hematocrit (0.33–0.47)	0.42 ± 0.02	0.41 ± 0.02	0.41 ± 0.03	0.868
Hemoglobin (115–160 g/L)	138.8 ± 8.79	138.3 ± 10.87	138.6 ± 13.87	0.882

Data presented as mean ± SD. Reference ranges for full blood count parameters have been included in the table. Values of p < 0.05 are in boldface. HC, healthy controls; ME/CFS, myalgic encephalomyelitis/chronic fatigue syndrome; SF-36, 36-item short form health survey; WHO, World Health Organization; DAS, disability assessment schedule.

**Table 3:** Symptom characteristics.

		ME/CFS	Post-COVID-19 condition
Age of diagnosis (years [mean ± SD])		23.78 ± 9.35	40.11 ± 11.14
Disease duration (years [mean ± SD])		15.56 ± 9.67	0.37 ± 0.14
Infectious onset, *N* (%)		5 (55.6%)	9 (100.0%)
Cognitive difficulties	Yes	9 (100.0%)	7 (77.8%)
	No	0 (0.0%)	2 (22.2%)
Pain	Yes	9 (100.0%)	8 (88.9%)
	No	0 (0.0%)	1 (11.1%)
Sleep disturbances	Yes	9 (100.0%)	8 (88.9%)
	No	0 (0.0%)	1 (11.1%)
Sensory disturbances	Yes	7 (77.8%)	8 (88.9%)
	No	2 (22.2%)	1 (11.1%)
Immune disturbances	Yes	7 (77.8%)	7 (77.8%)
	No	2 (22.2%)	2 (22.2%)
Gastrointestinal disturbances	Yes	8 (88.9%)	6 (66.7%)
	No	1 (11.1%)	3 (33.3%)
Cardiovascular disturbances	Yes	8 (88.9%)	7 (77.8%)
	No	1 (11.1%)	2 (22.2%)
Respiratory disturbances	Yes	6 (66.7%)	5 (55.6%)
	No	3 (33.3%)	4 (44.4%)
Thermostatic instability	Yes	6 (66.7%)	6 (66.7%)
	No	3 (33.3%)	3 (33.3%)
Urinary disturbances	Yes	6 (66.7%)	1 (11.1%)
	No	3 (33.3%)	8 (88.9%)

Data presented as mean ± SD and N (%). HC, healthy controls; ME/CFS, myalgic encephalomyelitis/chronic fatigue syndrome.

### 3.2 TRPM3 after PregS in freshly isolated NK cells (baseline)

**Figure 1:**
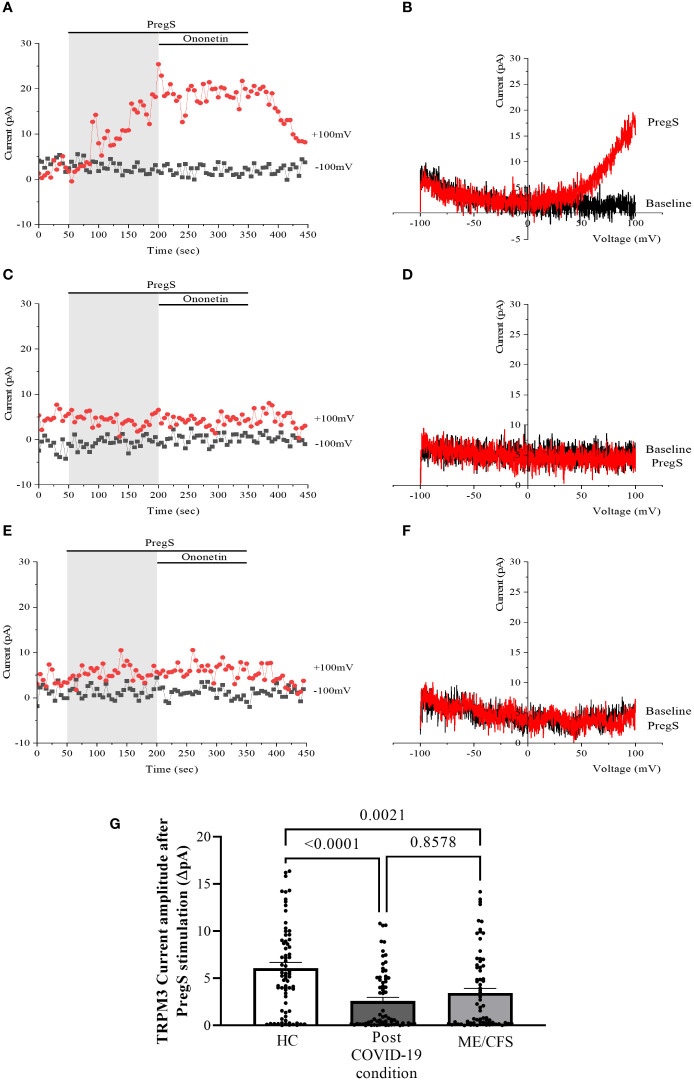
TRPM3 activity after PregS stimulation in freshly isolated NK cells. Data were obtained under whole-cell patch-clamp conditions. **(A)** A representative time series of current amplitude at +100 mV and −100 mV showing the effect of 100 µM PregS on ionic currents in freshly isolated NK cells from HC. **(B)** *I*–*V* before and after PregS stimulation in a cell corresponding to **(A)**. **(C)** A representative time series of current amplitude at +100 mV and −100 mV showing the effect of 100 µM PregS on ionic currents in freshly isolated NK cells from ME/CFS patients. **(D)**. *I*–*V* before and after PregS stimulation in a cell as shown in **(C)**. **(E)** A representative time series of current amplitude at +100 mV and −100 mV showing the effect of 100 µM PregS on ionic currents in freshly isolated NK cells from post-COVID-19 condition patients. **(F)** *I*–*V* before and after PregS stimulation in a cell corresponding to **(E)**. **(G)** Bar graphs representing TRPM3 current amplitude at +100 mV after stimulation with 100 µM PregS in HC patients (*N* = 9; *n* = 74) compared with post-COVID-19 condition patients (*N* = 9; *n* = 72) and ME/CFS patients (*N* = 9; *n* = 74). *N* refers to the number of participants, and *n* refers to the number of records analyzed. TRPM3 currents were determined as a change in amplitude from baseline to PregS-induced peak as represented in time-series graphs. *I*–*V* curves were used to identify an outward rectification typical of TRPM3. Data are presented as mean ± SEM. HC, healthy controls; ME/CFS, myalgic encephalomyelitis/chronic fatigue syndrome; PregS, pregnenolone sulfate; TRPM3, transient receptor potential melastatin 3.

Recordings of TRPM3 ion channel currents were obtained from freshly isolated NK cells from HC, post-COVID-19 condition patients, and ME/CFS patients using the whole-cell patch-clamp electrophysiological technique. Endogenous TRPM3 function was rapidly and reversibly activated by application of 100 μM PregS. We found a significant difference among three groups of ionic current amplitude after PregS stimulation (*p* < 0.0001). PregS stimulation enabled measurement of a small outwardly rectifying current under voltage-clamp conditions and a typical current–voltage relationship (*I*–*V*) shape of TRPM3. Small ionic currents with a typical TRPM3-outward rectification were measured in NK cells isolated from HC after PregS stimulation (**Figures 1A, B**). As widely characterized (53), NK cells from ME/CFS patients showed significantly smaller amplitude of the ionic currents after PregS stimulation (**Figures 1C, D**) than those from HC (**Figure 1G**, *p* = 0.0021). As we recently reported (53), PregS induced TRPM3 currents were significantly reduced in NK cells from the post-COVID-19 condition group (**Figures 1E, F**) compared with those from HC (**Figure 1G**, p < 0.0001), and were not significantly reduced from the ME/CFS group (**Figure 1G**, p = 0.8578).

### 3.3 Modulation with ononetin in freshly isolated NK cells (baseline)

**Figure 2:**
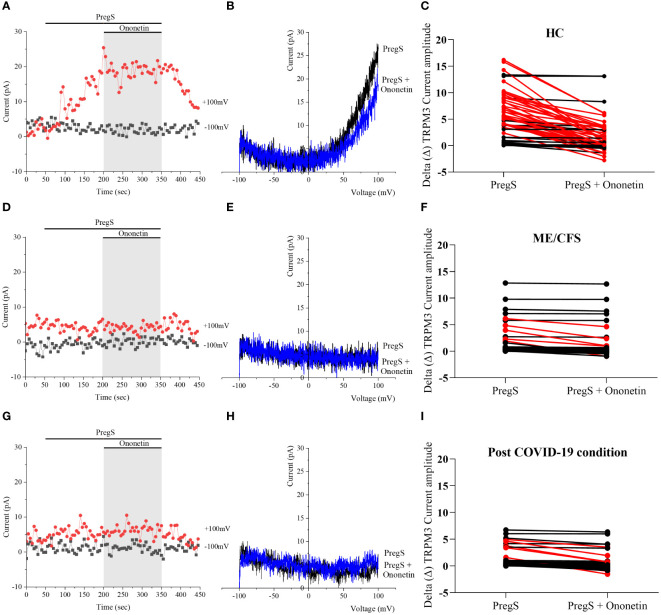
TRPM3 activity after ononetin modulation in freshly isolated NK cells. Data were obtained under whole-cell patch-clamp conditions. **(A)** A representative time series of current amplitude at +100 mV and −100 mV showing the effect of 10 µM ononetin on currents in the presence of PregS in freshly isolated NK cells from HC. **(B)** *I*–*V* before and after the application of ononetin in a cell as shown in **(A)**. **(C)** Scatter plots representing the change of each current amplitude before and after the application of ononetin in the presence of PregS in NK cells from HC. **(D)** A representative time series of current amplitude at +100 mV and −100 mV showing the effect of 10 µM ononetin on currents in the presence of PregS in freshly isolated NK cells from ME/CFS. **(E)** *I*–*V* before and after the application of ononetin in a cell as shown in **(D)**. **(F)** Scatter plots representing the change of each current amplitude before and after the application of ononetin in the presence of PregS in NK cells from ME/CFS. **(G)** A representative time series of current amplitude at +100 mV and −100 mV showing the effect of 10 µM ononetin on currents in the presence of PregS in freshly isolated NK cells from post-COVID-19 condition patients. **(H)** *I*–*V* before and after the application of ononetin in a cell as shown in **(G)**. **(I)** Scatter plots representing the change of each current amplitude before and after the application of ononetin in the presence of PregS in NK cells from post-COVID-19 condition patients. Each red line represented a cell sensitive to ononetin as a reduction in amplitude. HC (*N* = 9; *n* = 61), post-COVID-19 condition (*N* = 9; *n* = 47), and ME/CFS (*N* = 9; *n* = 47). *N* refers to the number of participants, and *n* refers to the number of records analyzed. HC, healthy controls; ME/CFS, myalgic encephalomyelitis/chronic fatigue syndrome; PregS, pregnenolone sulfate; TRPM3, transient receptor potential melastatin 3.

**Figure 3:**
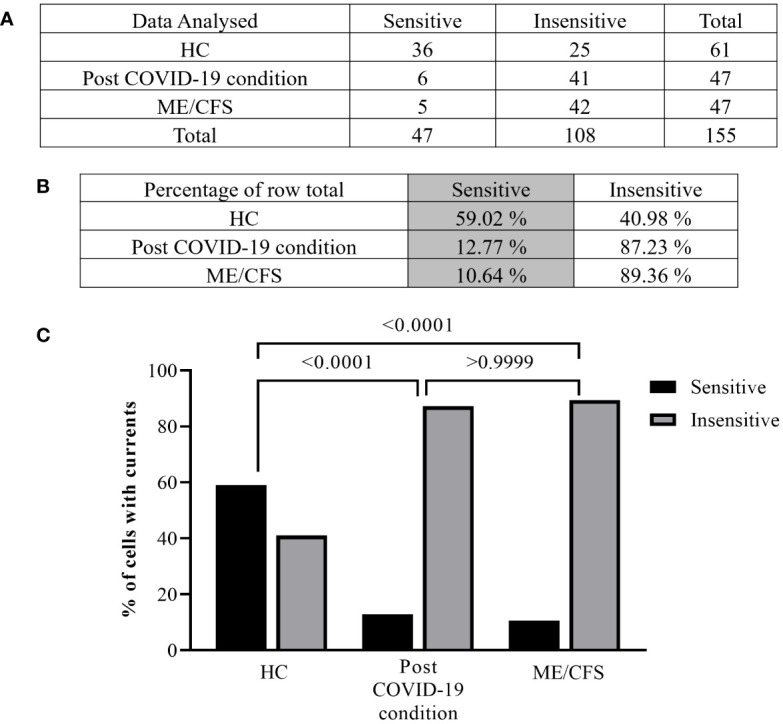
Summary TRPM3 activity after ononetin modulation in freshly isolated NK cells. Data were obtained under whole-cell patch-clamp conditions. Tables summarizing data for sensitive and insensitive cells to 10 µM ononetin, **(A)** absolute number and **(B)** percentage. **(C)** Bar graphs representing the percentage of sensitive and insensitive cells to 10 µM ononetin. HC (*N* = 9; *n* = 61), post-COVID-19 condition (*N* = 9; *n* = 47), and ME/CFS (*N* = 9; *n* = 47). *N* refers to the number of participants, and *n* refers to the number of records analyzed. Data were analyzed using Fisher’s exact test (applying Bonferroni method). HC, healthy controls; ME/CFS, myalgic encephalomyelitis/chronic fatigue syndrome.

Ca^2+^-influx and ionic currents through TRPM3 channels are induced by PregS application, while ononetin inhibits PregS-evoked currents (64). In this study, ononetin (10 μM) was applied in bath solution to modulate TRPM3 function and confirm that TRPM3 is involved in ionic currents previously stimulated by PregS (**Figures 2**, **3**). As demonstrated previously (31, 48, 49, 53), the PregS-evoked currents were blocked by simultaneous modulation with ononetin in cells from HC (**Figures 2A–C**), and the *I*–*V* of ononetin-sensitive currents showed an outward rectification and a typical shape for TRPM3 (**Figure 2B**). In contrast, in the presence of PregS, ionic currents were mostly resistant to ononetin in NK cells from ME/CFS patients (**Figures 2D–F**) and post-COVID-19 condition patients (**Figures 2G–I**), and the *I*–*V*s of ononetin currents in both groups did not show outwardly rectified and TRPM3-like currents (**Figures 2E, H**).

Furthermore, freshly isolated NK cells from ME/CFS and post-COVID-19 condition participants demonstrated resistance to ononetin application (89.36% and 87.23%, respectively) and were significantly different from those from HC (40.98%) (**Figures 3B, C**, *p* < 0.0001 and *p* < 0.0001). There was no significant difference identified between post-COVID-19 condition and ME/CFS (**Figure 3C**, *p* > 0.9999). Complementarily, in **Figures 2C, F, I**, scatter plots represented individual current changes before and after ononetin application, supporting the higher resistance of NK cells from ME/CFS and post-COVID-19 condition cells to ononetin.

### 3.4 TRPM3 after PregS in non-treated NK cells

**Figure 4:**
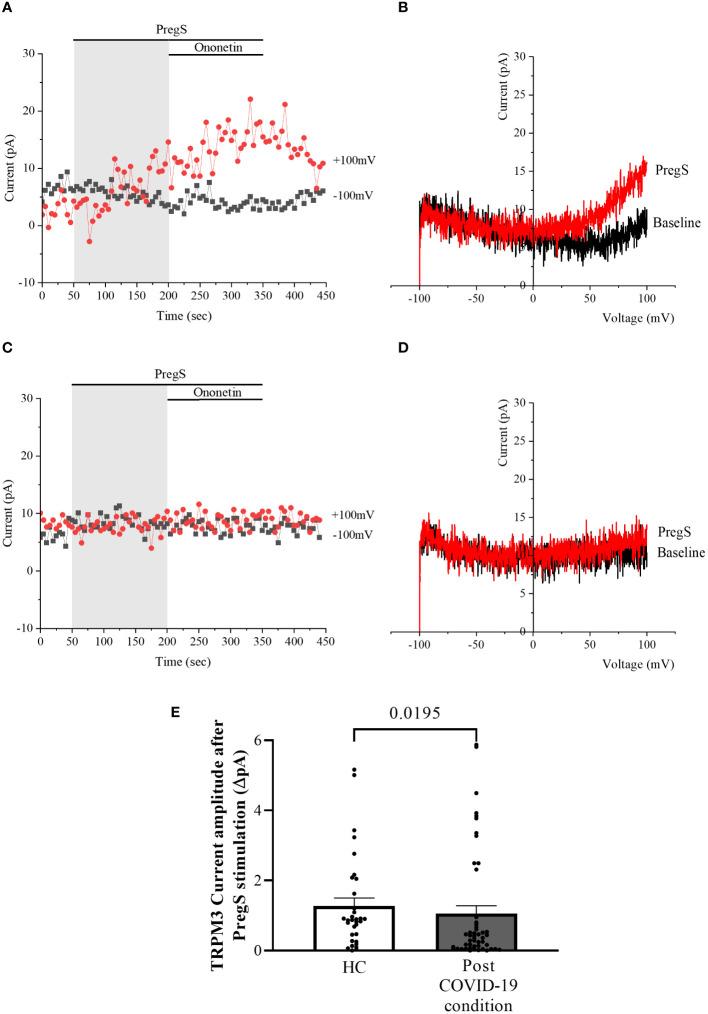
TRPM3 activity after PregS stimulation in non-treated NK cells. Data were obtained under whole-cell patch-clamp conditions. **(A)** A representative time series of current amplitude at +100 mV and −100 mV showing the effect of 100 µM PregS on ionic currents in non-treated NK cells from HC. **(B)** *I*–*V* before and after PregS stimulation in a cell corresponding to **(A)**. **(C)** A representative time series of current amplitude at +100 mV and −100 mV showing the effect of 100 µM PregS on ionic currents in non-treated NK cells from post-COVID-19 condition patients. **(D)**. *I*–*V* before and after PregS stimulation in a cell as shown in **(C)**. **(E)** Bar graphs representing TRPM3 current amplitude at +100 mV after stimulation with 100 µM PregS in HC patients (*N* = 7; *n* = 33) compared with post-COVID-19 condition patients (*N* = 9; *n* = 50). *N* refers to the number of participants, and *n* refers to the number of records analyzed. TRPM3 currents were determined as a change in amplitude from baseline to PregS-induced peak as represented in time-series graphs. *I*–*V* curves were used to identify an outward rectification typical of TRPM3. Data are presented as mean ± SEM. HC, healthy controls; PregS, pregnenolone sulfate; TRPM3, transient receptor potential melastatin 3.

TRPM3 function in non-treated NK cells from post-COVID-19 condition and HC groups was assessed in the whole-cell mode after 24 h of incubation without NTX as a control. As performed in freshly isolated NK cells (baseline), 100 μM PregS was applied to activate endogenous TRPM3 currents. In non-treated NK cells from HC, PregS evoked tiny outwardly rectifying currents under voltage-clamp conditions (**Figures 4A, B**), while typical TRPM3-like outward rectification was never observed in non-treated NK cells from post-COVID-19 condition patients (**Figures 4C, D**). Although the amplitude of ionic currents following PregS stimulation was small after each incubation, non-treated NK cells from post-COVID-19 condition patients had significantly smaller PregS-induced TRPM3-like currents (**Figure 4E**, *p* = 0.0195). Therefore, these results confirmed significant impairment of the TRPM3 channel in non-treated NK cells from post-COVID-19 condition patients.

### 3.5 Modulation with ononetin in non-treated NK cells

**Figure 5:**
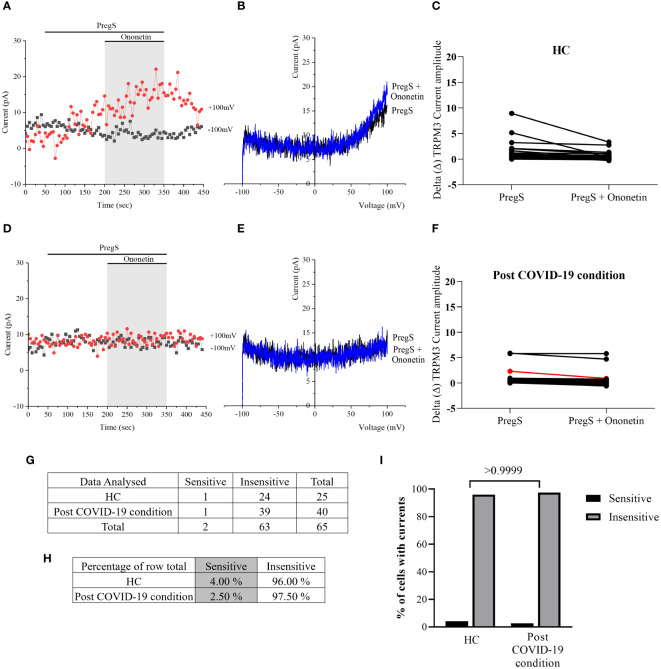
TRPM3 activity after ononetin modulation in non-treated NK cells. Data were obtained under whole-cell patch-clamp conditions. **(A)** A representative time series of current amplitude at +100 mV and −100 mV showing the effect of 10 µM ononetin on ionic currents in the presence of PregS in non-treated NK cells from HC. **(B)** *I*–*V* before and after the application of ononetin in a cell as shown in **(A)**. **(C)** Scatter plots representing the change of each current amplitude before and after the application of ononetin in the presence of PregS in all NK cells from HC. **(D)** A representative time series of current amplitude at +100 mV and −100 mV showing the effect of 10 µM ononetin on ionic currents in the presence of PregS in non-treated NK cells from post-COVID-19 condition patients. **(E)** *I*–*V* before and after the application of ononetin in a cell as shown in **(D)**. **(F)** Scatter plots representing the change of each current amplitude before and after the application of ononetin in the presence of PregS in all NK cells from post-COVID-19 condition patients. **(G)** Absolute number of sensitive and insensitive cells to 10 µM ononetin; **(H)** percentage of cells sensitive and insensitive to 10 µM ononetin. **(I)** Bar graphs representing percentage of sensitive and insensitive cells. HC (*N* = 7; *n* = 25) and post-COVID-19 condition patients (*N* = 9; *n* = 40). *N* refers to the number of participants, and *n* refers to the number of records analyzed. Each red line represented a cell sensitive to ononetin as a reduction in amplitude. Data were analyzed using Fisher’s exact test (applying Bonferroni method). HC, healthy controls; PregS, pregnenolone sulfate; TRPM3, transient receptor potential melastatin 3.

Despite significant difference in PregS-induced TRPM3-like currents between post-COVID-19 condition and HC groups (**Figure 4E**, *p* = 0.0195), no significant difference in the number of cells resistant to ononetin was shown between both groups (**Figure 5I**, *p* > 0.9999), suggesting that in non-treated NK cells, ononetin was ineffective to inhibit PregS-induced ionic currents through TRPM3 after 24 h of incubation. Furthermore, the *I*–*V* of ononetin-sensitive currents did not have clear outward rectification in NK cells from HC (**Figures 5A–C**) and post-COVID-19 condition patients (**Figures 5D–F**). Scatter plots in **Figures 5C, F** also represented the small difference in current amplitude before and after ononetin.

### 3.6 TRPM3 after PregS in NK cells after NTX treatment

**Figure 6:**
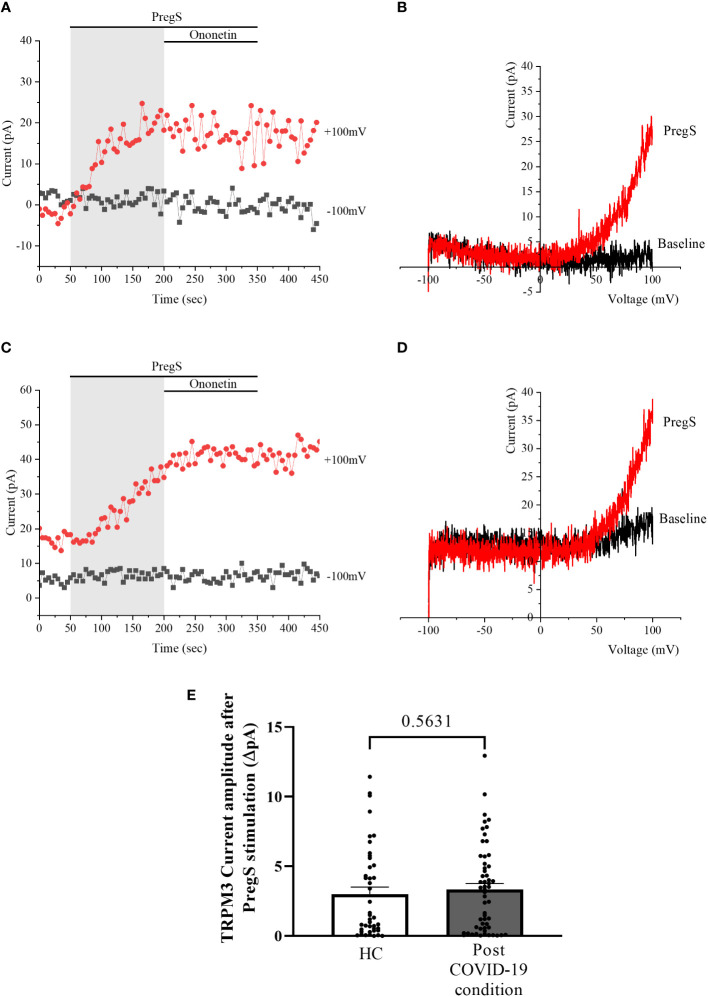
TRPM3 activity after PregS stimulation in NTX-treated NK cells. Data were obtained under whole-cell patch-clamp conditions. **(A)** A representative time series of current amplitude at +100 mV and −100 mV showing the effect of 100 µM PregS on ionic currents in NTX-treated NK cells from HC. **(B)** *I*–*V* before and after PregS stimulation in a cell corresponding to **(A)**. **(C)** A representative time series of current amplitude at +100 mV and −100 mV showing the effect of 100 µM PregS on ionic currents in NTX-treated NK cells from post-COVID-19 condition patients. **(D)**. *I*–*V* before and after PregS stimulation in a cell as shown in **(C)**. **(E)** Bar graphs representing TRPM3 current amplitude at +100 mV after stimulation with 100 µM PregS in HC patients (*N* = 7; *n* = 43) compared with post-COVID-19 condition patients (*N* = 9; *n* = 56). *N* refers to the number of participants, and *n* refers to the number of records analyzed. TRPM3 currents were determined as a change in amplitude from baseline to PregS-induced peak as represented in time-series graphs. *I*–*V* curves were used to identify an outward rectification typical of TRPM3. Data are presented as mean ± SEM. HC, healthy controls; PregS, pregnenolone sulfate; TRPM3, transient receptor potential melastatin 3.

After 24-h treatment with 200 μM NTX, whole-cell patch-clamp experiments were performed to assess TRPM3 activity in NK cells from post-COVID-19 condition and HC participants. PregS (100 μM) was again used to stimulate endogenous TRPM3 as performed in freshly isolated NK cells (baseline) and non-treated groups. In NTX-treated NK cells from HC, PregS induced small outwardly rectifying currents under voltage-clamp conditions and the typical TRPM3-like *I*–*V* was observed (**Figures 6A, B**), similar to freshly isolated NK cells from HC (baseline) (**Figures 1A, B**) and also non-treated NK cells (**Figures 4A, B**). In contrast to results of NK cells freshly isolated from post-COVID-19 condition (baseline) (**Figures 1E, F**) and non-treated (**Figures 4C, D**), NTX-treated NK cells from post-COVID-19 condition had PregS-evoked small outwardly rectifying currents under voltage-clamp conditions and a TRPM3-like *I*–*V* (**Figures 6C, D**), with no significant difference from NTX-treated NK cells from HC (**Figure 6E**, *p* = 0.5631). Interestingly, the post-COVID-19 condition group, after 24-h *in vitro* treatment with 200 µM NTX mimicked PregS-induced currents in NTX-treated NK cells from HC.

### 3.7 Modulation with ononetin in NK cells after NTX treatment

**Figure 7:**
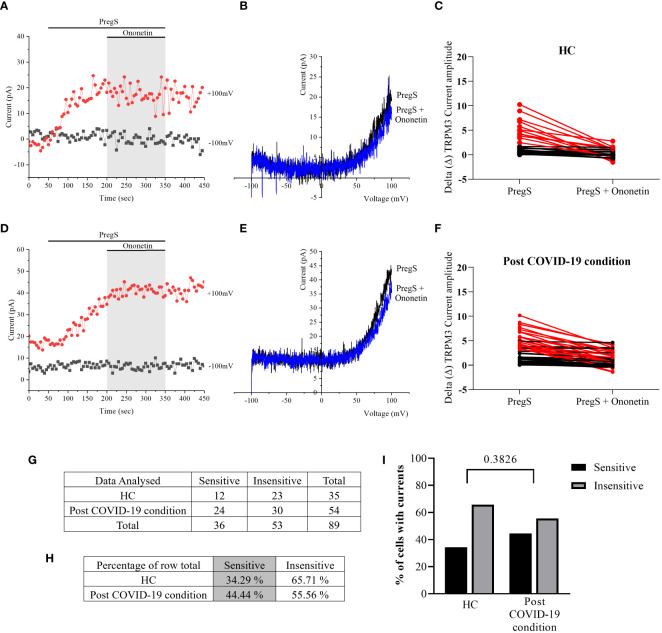
TRPM3 activity after ononetin modulation in NTX-treated NK cells. Data were obtained under whole-cell patch-clamp conditions. **(A)** A representative time series of current amplitude at +100 mV and −100 mV showing the effect of 10 µM ononetin on ionic currents in the presence of PregS in NTX-treated NK cells from HC. **(B)** *I*–*V* before and after the application of ononetin in a cell as shown in **(A)**. **(C)** Scatter plots representing the change of each current amplitude before and after the application of ononetin in the presence of PregS in all NK cells from HC. **(D)** A representative time series of current amplitude at +100 mV and −100 mV showing the effect of 10 µM ononetin on ionic currents in the presence of PregS in NTX-treated NK cells post-COVID-19 condition patients. **(E)** *I*–*V* before and after the application of ononetin in a cell as shown in **(D)**. **(F)** Scatter plots representing the change of each current amplitude before and after the application of ononetin in the presence of PregS in all NK cells from post-COVID-19 condition patients. **(G)** Absolute number of sensitive and insensitive cells to 10 µM ononetin; **(H)** percentage of cells sensitive and insensitive to 10 µM ononetin. **(I)** Bar graphs representing percentage of sensitive and insensitive cells. HC (*N* = 7; *n* = 35) and post-COVID-19 condition patients (*N* = 9; *n* = 54). *N* refers to the number of participants, and *n* refers to the number of records analyzed. Each red line represented a cell sensitive to ononetin as a reduction in amplitude. Data were analyzed using Fisher’s exact test (applying Bonferroni method). HC, healthy controls; PregS, pregnenolone sulfate; TRPM3, transient receptor potential melastatin 3.

**Figure 8:**
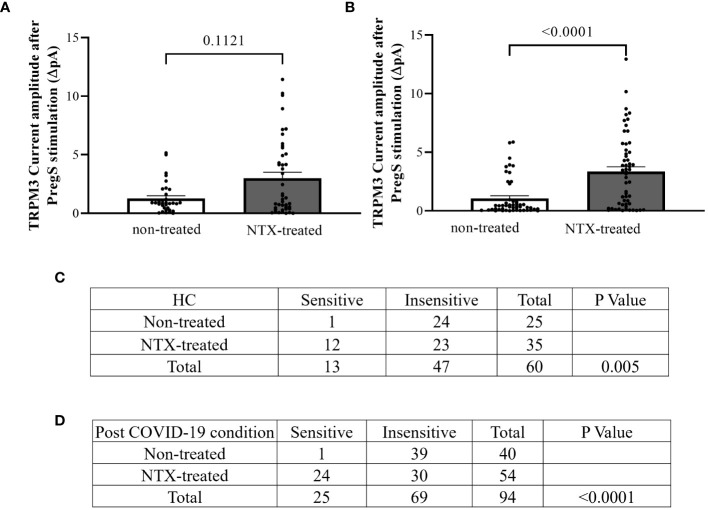
TRPM3 activity after PregS stimulation and ononetin modulation, comparing non-treated and NTX-treated NK cells. Data were obtained under whole-cell patch-clamp conditions. **(A)** Bar graphs representing TRPM3 current amplitude at +100 mV after stimulation with 100 µM PregS in NK cells from HC (*N* = 7) comparing non-treated (*n* = 33) with NTX-treated (*n* = 43). **(B)** Bar graphs representing TRPM3 current amplitude at +100 mV after stimulation with 100 µM PregS in NK cells from post-COVID-19 condition patients (*N* = 9) comparing non-treated (*n* = 50) with NTX-treated (*n* = 56). **(C)** Table summarizing data for sensitive and insensitive cells to 10 µM ononetin from HC (*N* = 7): comparison of non-treated (*n* = 25) with NTX-treated NK cells (*n* = 35). **(D)** Table summarizing data for sensitive and insensitive cells to 10 µM ononetin from post-COVID-19 condition patients (*N* = 9): comparison of non-treated (*n* = 40) with NTX-treated NK cells (*n* = 54). *N* refers to the number of participants, and *n* refers to the number of records analyzed. Ononetin data were analyzed using Fisher’s exact test (applying Bonferroni method). Data are presented as mean ± SEM. HC, healthy controls; NTX, naltrexone hydrochloride; PregS, pregnenolone sulfate; TRPM3, transient receptor potential melastatin 3.

In NTX-treated NK cells, ononetin was applied to confirm the involvement of the TRPM3 channel function in ionic currents evoked by PregS application (**Figure 7**). Although the currents of NK cells from post-COVID-19 condition were mostly resistant to ononetin in freshly isolated (baseline) (**Figures 2**, **3**) and non-treated cells (**Figure 5**), *in vitro* treatment with 200 μM NTX in part restored the sensitivity to ononetin (**Figures 7**, **8**). In NTX-treated NK cells from the post-COVID-19 condition group, *I*–*V* of ononetin-sensitive currents was outwardly rectified (**Figure 7E**), with no significant difference of the resistance to ononetin from HC (**Figure 7I**, *p* = 0.383). Meanwhile, when PregS-induced TRPM3 currents were compared between non-treated and NTX-treated NK cells from HC, no significant difference was found (**Figure 8A**, *p* = 0.1121). However, after the same incubation processes of NK cells from the post-COVID-19 condition group, TRPM3 currents were significantly increased in the NTX-treated group (**Figure 8B**, *p* < 0.0001). Sensitivity to ononetin of NK cells was also significantly changed in NTX-treated NK cells from HC and post-COVID-19 condition participants (**Figures 8C, D**, *p* = 0.005 and *p* < 0.0001, respectively). Our results suggest that TRPM3 function in NK cells from post-COVID-19 condition participants was restored after *in vitro* treatment with 200 μM NTX.

## 4 Discussion

Our previous research has suggested that people suffering from post-COVID-19 condition also have TRPM3 dysfunction in NK cells (53), which is a biomarker consistently identified in ME/CFS patients (31, 48, 49). Subsequently, we proposed in the present study to achieve two different aims: (i) validate impaired TRPM3 ion channel function in post-COVID-19 condition compared with ME/CFS using fresh isolated cells (baseline), and (ii) investigate NTX effects on TRPM3 channel function in NK cells from post-COVID-19 condition participants after incubation of non-treated and NTX-treated cells.

This investigation further supports the hypothesis of ion channel dysfunction overlapping between both illnesses. Indeed, a significant reduction in TRPM3 amplitude after PregS (100 µM) application was shown in freshly isolated NK cells from post-COVID-19 condition participants compared with cells from the HC group (**Figure 1G**, *p* < 0.0001). In addition, TRPM3 ion channel currents from post-COVID-19 condition participants were resistant to 10 µM ononetin unlike those from HC (**Figure 3C**, *p* < 0.0001). In contrast, TRPM3 currents from the post-COVID-19 condition group were similar to those from the ME/CFS group: both groups had tiny PregS-induced TRPM3 currents and resistance to ononetin (**Figure 1G**, *p* = 0.8578; **Figure 3C**, *p* > 0.9999), strongly suggesting that ME/CFS and post-COVID-19 condition are associated with impaired TRPM3 function in NK cells.

This study showed that TRPM3 ion channel activity was restored in NK cells from post-COVID-19 condition patients after *in vitro* treatment with 200 μM NTX for 24 h. Additionally, NTX-treated NK cells from post-COVID-19 condition patients in part became sensitive to ononetin when compared with non-treated cells (**Figure 8D**, *p* < 0.0001), confirming that TRPM3 ion channel currents were evoked by PregS stimulation in NTX-treated NK cells from post-COVID-19. Importantly, PregS-induced current amplitude (**Figure 6E**, *p* = 0.5631) and the sensitivity of the currents to ononetin (**Figure 7I**, *p* = 0.3826) were not different between HC and post-COVID-19 following NTX treatment of NK cells. The design of this study included a control group for incubation to avoid bias, comparing results from NTX-treated NK cells with the new baseline (non-treated), ensuring analysis is between cells exposed to the same conditions, except NTX. No significant differences in PregS-induced TRPM3 ionic currents were found in HC between NTX-treated and non-treated cells (**Figure 8A**, *p* = 0.1121), confirming TRPM3 function after incubation. Overall, results from this current investigation demonstrate restoration of TRPM3 activity in NK cells from post-COVID-19 condition participants.

Dysregulation of TRPM3 affects intracellular Ca^2+^ concentration level, which potentially may impair cell function and intracellular signaling pathways (31, 34, 48). In particular, Ca^2+^ signaling is impacted by TRPM3 impairment in ME/CFS patients, and it consequently causes a dysregulation on NK cells (31, 49). Furthermore, as TRPM3 on these cells contribute to the Ca^2+^-dependent phosphorylation of signaling proteins [phosphoinositide 3-kinase (PI3K) and mitogen-activated protein kinases (MAPK)], it may affect NK cell function, such as cytotoxic function and cytokine production (65). In fact, live-cell immunofluorescent imaging study using NK cells from ME/CFS patients revealed a significant decrease in Ca^2+^ influx via TRPM3 (34).

NTX is a non-selective opioid antagonist with activity at several opioid and non-opioid human receptors. As an μOR antagonist, NTX negates the inhibitory function of μOR on TRPM3 ion channels, whereas TRPM3 is inhibited by GPCRs through direct binding of Gβγ proteins and the overexpression of Gβγ proteins inhibits TRPM3 (66–69). In particular, TRPM3 is inhibited by activation of three GPCRs, G_i_-coupled µOR, γ-aminobutyric acid type B (GABA-B), and neuropeptide Y, in mouse dorsal root ganglion (DRG) neurons (67–69). Morphine, a strong opioid prescribed to relieve severe pain, inhibits TRPM3 by activating ORs in DRG neurons. Although morphine is a non-selective OR agonist, Quallo et al. identified that TRPM3 is specifically inhibited by µOR activation, testing different OR subtype agonists. Notably, TRPM3 channel activity in somatosensory neurons regulated by μOR reduces TRPM3-dependent pain (70).

Interestingly, this current investigation reports, for the first time, the restoration of the TRPM3 ion channel function with *in vitro* NTX treatment in NK cells from post-COVID-19 condition participants, similar to previous results shown in ME/CFS by Cabanas et al. (49). The NTX restoration of dysfunctional TRPM3 may recover cellular Ca^2+^-dependent mechanisms, such as the integrity and stability of NK cell-specific signaling systems (49). Importantly, NTX restored TRPM3 in NK cells from both post-COVID-19 condition and ME/CFS groups, suggesting TRPM3 dysfunction overlap in the pathomechanism of both conditions. The results from this investigation therefore support the potential application of NTX as a pharmacotherapeutic treatment in ME/CFS and post-COVID-19 patients.

Similar results of *in vitro* NTX on TRPM3 restoration were shown in NK cells from ME/CFS patients taking LDN, and TRPM3 currents were restored in these cells (56). Indeed, LDN was indicated as having a potential benefit for the treatment of ME/CFS patients, as well as other debilitating diseases such as fibromyalgia, multiple sclerosis, Crohn’s disease, and cancer (56, 71–79). Polo et al. reported that 73.9% of ME/CFS patients taking LDN responded positively to treatment, including improvement in vigilance, as well as physical and cognitive performance that are frequent symptoms in post-COVID-19 condition (74). In another study, the benefits of LDN for post-COVID-19 condition patients included having a safety range of 94.7% and an improvement in six out of seven parameters measured, including recovery from COVID-19, limitation in activities of daily living, sleep disturbance, pain, and energy level concentration, while no improvement in mood was reported (11).

ME/CFS symptoms overlap with post-COVID-19 condition, such as significant negative impacts on QoL and people’s routine, which may include disruptions in work performance, social, or home activities (1, 5, 7, 10, 12, 24, 27, 80–82). Considering the prevalence of the post-COVID-19 condition, and that currently more than 768 million of SARS-CoV-2 infections were confirmed worldwide, this condition is a public health and economic concern and could be a highly prevalent long-term condition if no effective treatments are developed (6, 7). NTX pharmacology is well known and approved for the treatment of opioid and alcohol addiction and LDN is used as an off-label treatment for several disorders in many countries (68, 71–74). Thus, this provides an opportunity to pursue drug repurposing for the treatment of ME/CFS and post-COVID-19 condition.

In conclusion, TRPM3 dysfunction in post-COVID-19 condition and ME/CFS participants suggests impairment in ion mobilization and consequently results in Ca^2+^ signaling and cell homeostasis disturbance in both diseases. The NTX treatment restored TRPM3 ion channel activity in the post-COVID-19 condition group, facilitating Ca^2+^ influx for intracellular signaling pathways. Our data provide novel evidence for the usefulness of ion channel targeting pharmacotherapy using NTX and LDN. Further investigation will be required to evaluate the potential clinical application of LDN to post-COVID-19 condition patients such as assessing TRPM3 ion channel activity in NK cells from patients taking LDN, and clinical trials to confirm the benefit of LDN in improving QoL of post-COVID-19 condition patients.

## Data availability statement

The raw data supporting the conclusions of this article will be made available by the authors, without undue reservation.

## Ethics statement

The studies involving humans were approved by Griffith University Human Research Ethics Committee (GU HREC 2022/666). The studies were conducted in accordance with the local legislation and institutional requirements. The participants provided their written informed consent to participate in this study.

## Author contributions

EMS: Methodology, Investigation, Formal analysis, Data curation, Writing – original draft, Writing – review & editing, Conceptualization. KM: Methodology, Validation, Data curation, Writing – review & editing. NE-F: Methodology, Validation, Data curation, Writing – review & editing, Supervision, Conceptualization. PS: Writing – review & editing. AJ: Writing – review & editing. PG: Writing – review & editing. SM-G: Methodology, Validation, Data curation, Writing – review & editing, Supervision, Funding acquisition, Conceptualization.

## Supplementary material

Supplementary Table 1 Symptom presentation in post-COVID-19 condition and ME/CFS patients. Data presented as median. ME/CFS, myalgic encephalomyelitis/chronic fatigue syndrome.
