# Supplementary material for: Investigation into the restoration of TRPM3 ion channel activity in post-COVID-19 condition: a potential pharmacotherapeutic target
Source: Front Immunol. 2024 May 3;15:1264702. doi: 10.3389/fimmu.2024.1264702 (PMC11099221; doi:10.3389/fimmu.2024.1264702)
Supplement: Supplementary Table 1 — Symptom presentation in post-COVID-19 condition and ME/CFS patients. Data presented as median. ME/CFS, myalgic encephalomyelitis/chronic fatigue syndrome. [file Table_1.docx]

Supplementary Table 1: Symptom presentation in Post COVID-19 Condition and ME/CFS patients.

|  |  | Post COVID-19 Condition | ME/CFS |
| --- | --- | --- | --- |
| Fatigue |  |  |  |
|  | Frequency | Most of the time | All of the time |
|  | Severity | Moderate | Moderate |
| Concentration |  |  |  |
|  | Frequency | Some of the time | Most of the time |
|  | Severity | Mild | Moderate |
| Sleep Disturbances |  |  |  |
|  | Frequency | A good bit of the time | All of the time |
|  | Severity | Moderate | Moderate |
| Body Pain |  |  |  |
|  | Frequency | A good bit of the time | A good bit of the time |
|  | Severity | Moderate | Mild |
| Sensitivities to light |  |  |  |
|  | Frequency | Some of the time | A good bit of the time |
|  | Severity | Mild | Very mild |
| Dizziness, light headedness |  |  |  |
|  | Frequency | Some of the time | Some of the time |
|  | Severity | Mild | Mild |
| Respiratory |  |  |  |
|  | Frequency | A good bit of the time | A little of the time |
|  | Severity | Mild | Mild |
| Muscle weakness |  |  |  |
|  | Frequency | A good bit of the time | A good bit of the time |
|  | Severity | Moderate | Mild |
| Throat |  |  |  |
|  | Frequency | A little of the time | A little of the time |
|  | Severity | Mild | Very mild |

Data presented as median. Abbreviations: ME/CFS, Myalgic Encephalomyelitis/Chronic Fatigue Syndrome.
