# Supplementary figures and images for: Investigation into the restoration of TRPM3 ion channel activity in post-COVID-19 condition: a potential pharmacotherapeutic target
Source: Front Immunol. 2024 May 3;15:1264702. doi: 10.3389/fimmu.2024.1264702 (PMC11099221; doi:10.3389/fimmu.2024.1264702)

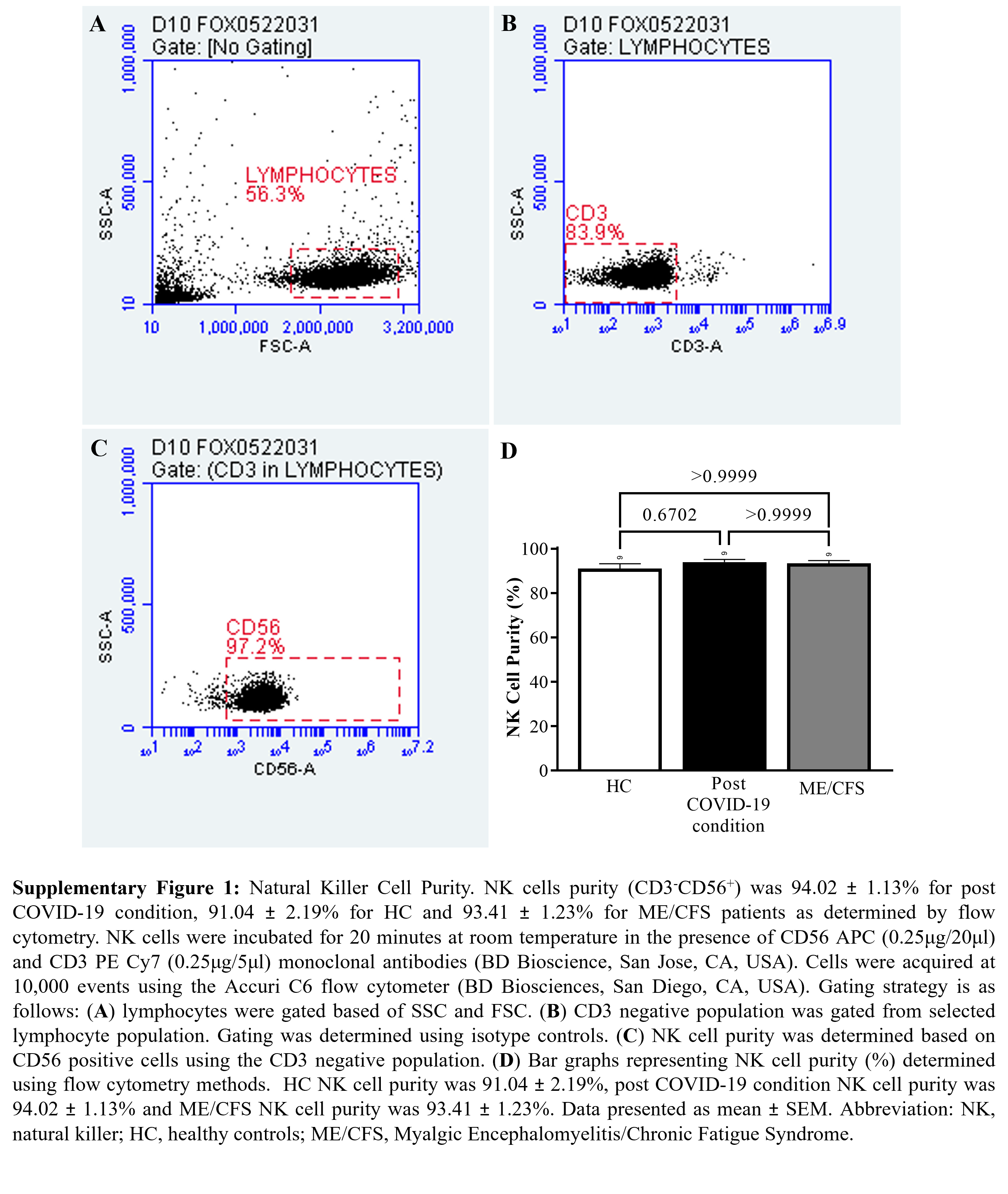

Supplement: Supplementary file 2 [file Image_1.tif]

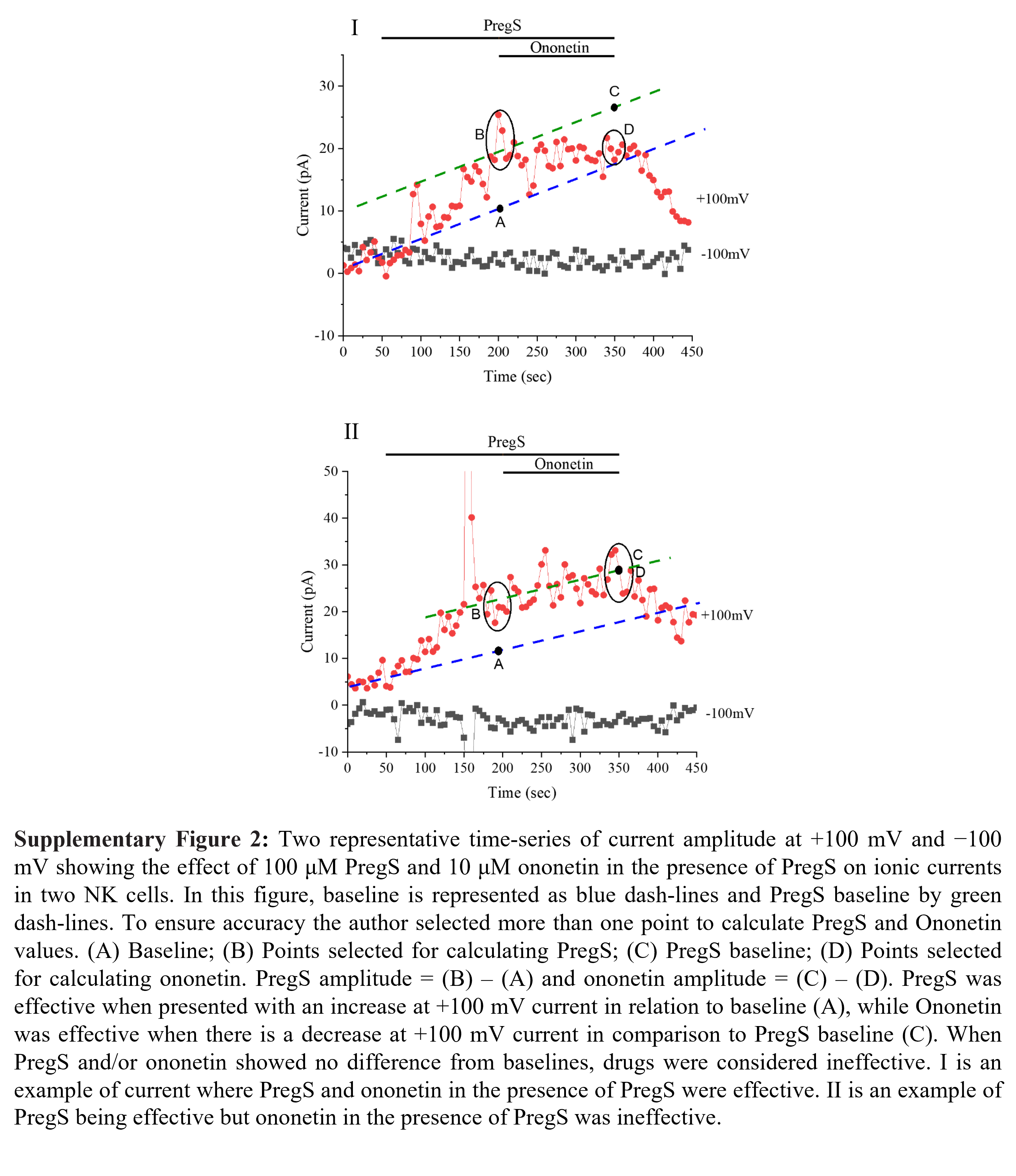

Supplement: Supplementary file 3 [file Image_2.tif]

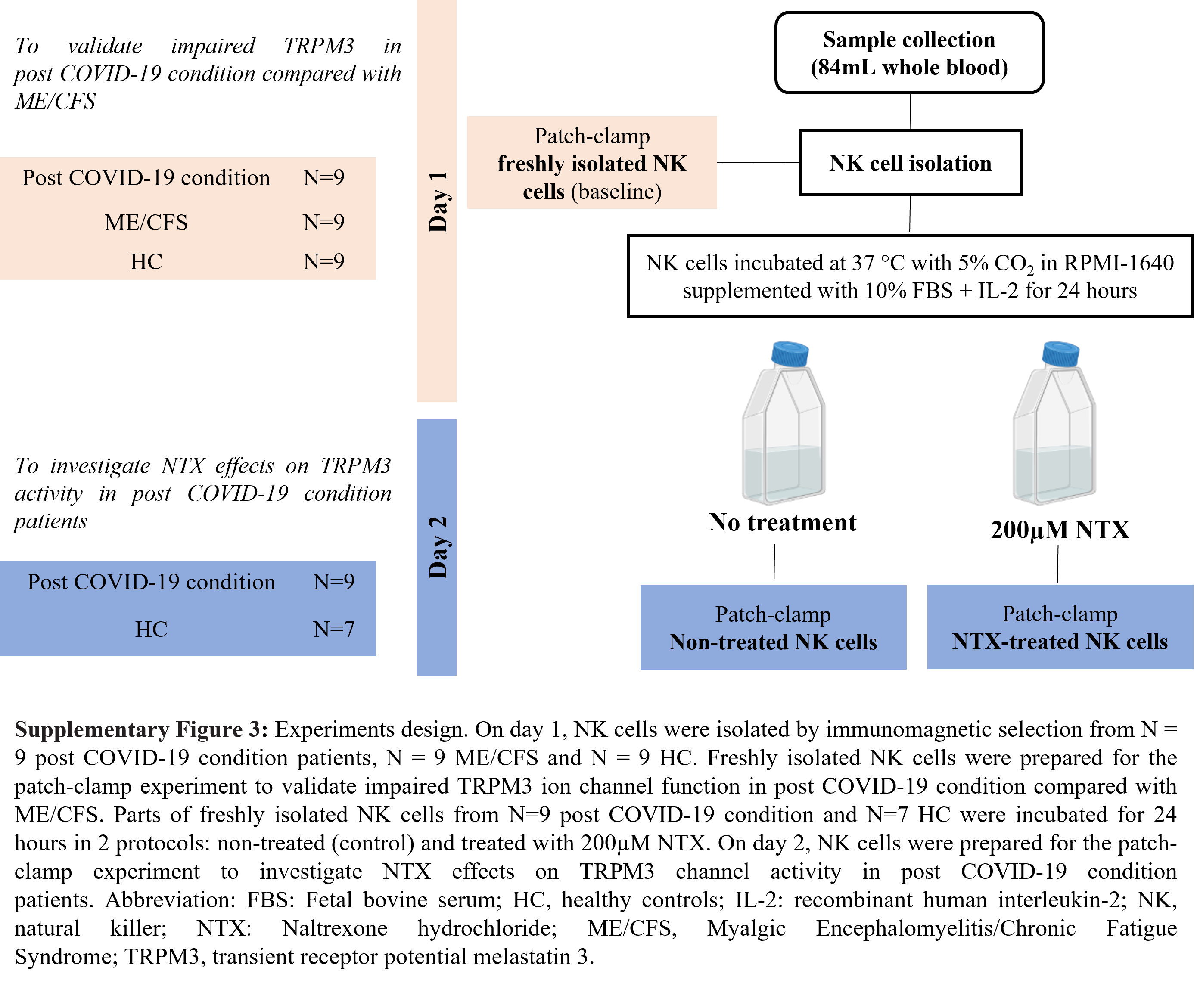

Supplement: Supplementary file 4 [file Image_3.tif]
